# Reply

**DOI:** 10.1002/uog.22105

**Published:** 2020-09-01

**Authors:** S. Hancock, R. Ben‐Shachar, C. Haverty, D. Muzzey

**Affiliations:** ^1^ Myriad Women's Health South San Francisco CA USA; ^2^ Myriad Genetics Salt Lake City UT USA

We are pleased that our recent study has generated interest in the scientific and clinical community, and we welcome the opportunity to further discuss its design and motivation^1^. We designed our study with the goal of presenting the expected performance of our custom whole‐genome sequencing (WGS)‐based non‐invasive prenatal screening (NIPS) approach in a real‐world clinical setting. To our knowledge, this study is novel in that we evaluated test performance across the entire spectrum of fetal fraction (FF) level and focused particularly on test performance in low‐FF samples (FF < 4%), which are often failed by other NIPS offerings. Jelsema *et al*. highlight several concerns regarding our recent publication which we wish to address.

The first main assertion by Jelsema *et al*. is that the outcome of all tested samples must be known in order to be able to calculate accurate test‐performance metrics. This assertion is inconsistent with the established convention among NIPS clinical‐experience papers^2^, ^3^. In our study, we assessed a prospectively identified clinical cohort of 58 105 singleton pregnancies which included challenging samples, such as those from mosaic or vanishing‐twin pregnancies. Given the large size of our cohort, we collected outcomes on a subset of these pregnancies, which is consistent with the approach in other clinical‐experience studies^2^, ^3^, ^4^. Furthermore, the rate of definitive‐outcome collection (based on karyotype or postnatal evaluation) for screen‐positive patients in our study is comparable to or higher than that in similar studies evaluating other NIPS tests^2^, ^3^, ^4^. Critically, instead of assuming that all screen‐negative results were true negatives^2^, ^3^, our study is one of the few USA‐based studies on clinical experience with NIPS to actively seek outcomes on screen‐negative results, successfully collecting outcomes from over 3000 pregnancies. Our study represents clinical practice to a greater extent than analytical validity studies, which reflect idealized test performance^5^. Analytical validity studies often exclude challenging samples and are typically small in order to allow procurement of complete outcomes^5^.

In addition to designing our study to comply with conventional approaches, we performed a host of additional analyses to assess the impact of our assumptions on the confidence in our results. One of the three methods that we used for this purpose is identical to the approach employed in many clinical‐experience studies, namely to assume that all missing outcomes are either true or false and recalculate each metric accordingly^3^, ^4^, ^6^. Another critical analysis showed that even if only 60% of outcomes of false‐negative samples (rather than 100%) were reported to our laboratory, the inferred sensitivity of our assay would change by less than 1%. Taken together, multiple analyses demonstrated that our test‐performance estimates are reflective of the capabilities of our custom WGS‐based NIPS approach as it applies to daily clinical practice.

The second main claim of Jelsema *et al*. is that our assay does not have comparable performance at low (< 4%) and high (≥ 4%) FF. However, the inferred sensitivity of our assay for the common aneuploidies was 95.7% (uncertainty range, 91.7–99.2%) for samples with FF < 4% and 98.9% (uncertainty range, 97.4–99.0%) for samples with FF ≥ 4%, as reported in table 3 of our paper^1^. There is clearly a high overlap in the uncertainty ranges for high and low FF. Given these and other extensive analyses described above, we stand by our assertion that our NIPS test has comparable performance at high and low FF. Moreover, it is important to put in context our findings: the lower bound of the uncertainty range of sensitivity for samples with FF < 4% is above 91%, whereas previously published modeling results suggest that the single‐nucleotide polymorphism platform has ∼ 80% sensitivity at 3% FF^7^. Thus, even if there is a small difference in the sensitivity of our assay between high and low FF, the magnitude of the difference is less than a level already deemed acceptable by other laboratories.

Jelsema *et al*. raise three additional minor points related to our study. First, they note that we should not state that our assay has a negative predictive value (NPV) of 100% for trisomy 13 in light of the two trisomy‐13 false‐negatives in our cohort. The unrounded NPV of our assay for trisomy 13 was 99.9965%, which we presented as 100% in the original version of the paper but we modified during proof checking to specify it is ‘> 99.99%’ (table 3)^1^. Second, the authors claim we have misrepresented data by rendering ‘likely’ negatives in figure 2 but not ‘likely’ positives. We displayed the data in this way because all false‐negative results in our study were self‐reported by clinicians, whereas we cannot be similarly confident that unconfirmed screen‐positive results are true positives. Therefore, in figure 2, we are being both transparent about our screen‐negative results (by distinguishing likely from confirmed results) and conservative about our screen‐positive results by showing only those that were confirmed. Finally, the authors note that our updated algorithm was applied *post hoc* to the study cohort. We are puzzled by this criticism because our approach is akin to the strategy used in a clinical‐experience study evaluating the performance of a NIPS assay for detecting microdeletions, which was conducted by Jelsema *et al*.'s laboratory^6^. Specifically, Martin *et al*. applied *post hoc* an updated algorithm to a cohort screened for microdeletions using NIPS, and quote the positive predictive values of the updated algorithm in the abstract and main text (e.g. figure 5) of the study^6^.

In conclusion, our study was designed to reflect the real‐world clinical performance of a custom WGS‐based NIPS approach that does not utilize a FF threshold. We were transparent and clear about our methods and results, performed a host of analyses to assess the significance of our results from different perspectives and discussed openly the limitations of our conclusions. Our finding that our assay performs well for both low‐ and high‐FF samples may serve to improve the patient and provider experience with NIPS. The anxiety and burden of test failures and redraws can be replaced by providing patients with accurate screening results the first time.
